# Prevalence and Characteristics of *Plasmodium vivax* Gametocytes in Duffy-Positive and Duffy-Negative Populations across Ethiopia

**DOI:** 10.4269/ajtmh.23-0877

**Published:** 2024-04-16

**Authors:** Ebony Little, Tassew T. Shenkutie, Meshesha Tsigie Negash, Beka R. Abagero, Abnet Abebe, Jean Popovici, Sindew Mekasha Feleke, Eugenia Lo

**Affiliations:** ^1^ Department of Biological Sciences, University of North Carolina at Charlotte, North Carolina;; ^2^ Department of Microbiology and Immunology, Drexel University, College of Medicine, Philadelphia, Pennsylvania;; ^3^ Department of Medical Laboratory Sciences, Debre Brehan University, Ethiopia;; ^4^ Ethiopian Public Health Institute, Addis Ababa, Ethiopia;; ^5^ Department of Molecular and Cellular Biology and Genetics, Drexel University, College of Medicine, Philadelphia, Pennsylvania;; ^6^ Institute Pasteur in Cambodia, Phnom Penh, Cambodia

## Abstract

*Plasmodium* parasites replicate asexually in human hosts. The proportion of infections that carries gametocytes is a proxy for human-to-mosquito transmissibility. It is unclear which proportion of *Plasmodium vivax* infections in Duffy-negative populations carries gametocytes. We determined the prevalence and characteristics of *P. vivax* gametocytes in Duffy-positive and -negative populations across broad regions of Ethiopia. Finger-prick blood samples were collected for microscopic and molecular screening of *Plasmodium* parasites and Duffy status of individuals. Molecular screening of *Plasmodium* species and Duffy blood group genotyping was done using SYBR green and the Taqman quantitative polymerase chain reaction method. Of the 447 febrile patients who were shown to be *P. vivax* smear positive, 414 (92.6%) were confirmed by molecular screening as *P. vivax* and 16 (3.9%) of them were from Duffy-negative individuals. Of these, 5 of 16 (31.3%) Duffy-negative *P. vivax*–infected samples were detected with gametocytes. Of the 398 Duffy-positive *P. vivax*–infected samples, 150 (37.7%) were detected with gametocytes, slightly greater than that in Duffy-negative samples. This study highlights the presence of *P. vivax* gametocytes in Duffy-negative infections, suggestive of human-to-mosquito transmissibility. Although *P. vivax* infections in Duffy-negative individuals were commonly associated with low parasitemia, some of these infections were shown to have relatively high parasitemia and may represent a prominent erythrocyte invasion capability of *P. vivax*, and hidden reservoirs that can contribute to transmission. A better understanding of *P. vivax* transmission biology and gametocyte function particularly in Duffy-negative populations would aid future treatment and management of *P. vivax* malaria in Africa.

## INTRODUCTION

Yearly, there are ∼619,000 malaria-related deaths and ∼247 million malaria cases reported globally.^1^ Of the five malaria *Plasmodium* species, *Plasmodium vivax* is the most widespread.^2^ Duffy-negative individuals were thought to be resistant to *P. vivax* infections. However, a growing number of *P. vivax* cases reported throughout Africa where Duffy-negative individuals predominate,^3^ demonstrated that *P. vivax* can infect Duffy-negative individuals,^4^^,^^5^ and could potentially spread and transmit across populations.^5^^,^^6^ As a result of epidemiological and ethnic differences, the prevalence of *P. vivax* in Duffy-negative individuals varies across Africa.^4^ Considering *P*. *vivax* can infect and adapt to Duffy-negative individuals, it is possible that these infections can produce gametocytes leading to transmission.^7^ The extent of transmission may vary by environmental and host factors.^8^

During the *Plasmodium* life cycle, the parasites undergo multiple asexual replicative cycles in the human host, and in each erythrocytic replication cycle, a small portion (∼0.1–5%) of the asexual stages develops into sexual gametocytes. The proportion of infections that carries gametocytes is a proxy for human-to-mosquito transmissibility.^9^ Within the mosquito midgut, male and female gametocytes undertake gametogenesis.^10^ After the gametes have fertilized, a zygote is created that later transforms into a motile ookinete. Under the basal lamina, ookinetes form an oocyst by crossing the midgut epithelium.^10^^,^^11^ Many thousands of sporozoites develop in the oocyst, and—as the oocyst wall ruptures—sporozoites enter the hemolymph and infect the salivary gland. The intricate life cycle of the parasite is then completed when sporozoites are inoculated into another person through mosquito bites.^12^ Gametocytogenesis is influenced by epigenetic, ecological, and heritable factors associated with the parasite.^13^ The occurrence of gametocytogenesis is also influenced by factors associated with human hosts, such as immunity status, antimalaria drug treatment, and genetic factors.^14^^,^^15^

The distribution of *P. vivax* in Duffy-negative individuals across Ethiopia, as well as the parasite stages of these infections, remain largely unclear. The presence of gametocytes in symptomatic or asymptomatic individuals can lead to onward transmission in communities.^16^ Knowledge of gametocyte reservoirs allows for prioritizing transmission-blocking vaccines against *P. vivax* in Africa.^17^^,^^18^ In this study, we 1) compared the distribution of *P. vivax* in Duffy-positive and Duffy-negative populations across Ethiopia, 2) determined the different stages of *P. vivax* in Duffy-positive and Duffy-negative infections, and 3) examined demographic and clinical features of Duffy-negative *P. vivax* infections. These findings advance current knowledge of *P. vivax* malaria distribution and transmission in Africa.

## MATERIALS AND METHODS

### Study sites.

**Figure 1.**
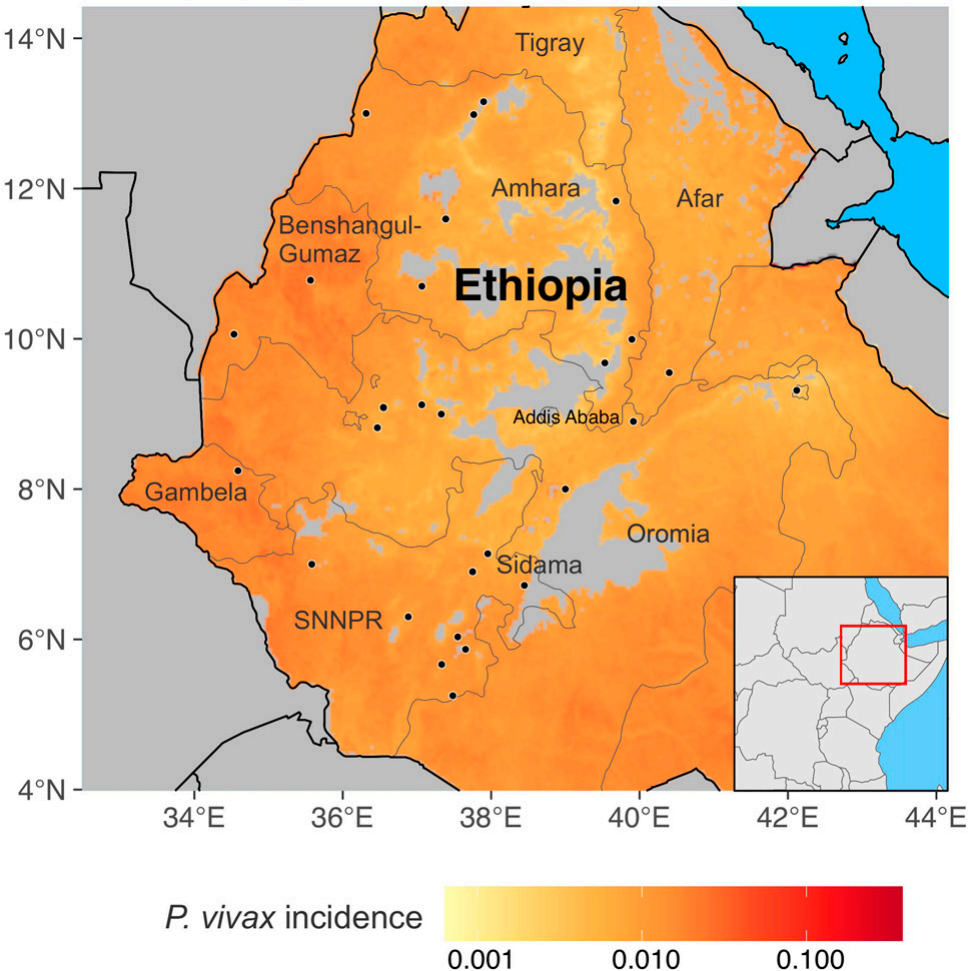
A map showing the study sites in Ethiopia with malaria incidence ranges from high in the western part to low in the eastern part of the country. These sites represent seven major regions: Afar, Amhara, Benishangul/Gumuz, Gambella, Oromia, Sidama, and Southern Nations, Nationalities, and People’s Region (SNNPR) with diverse ethnic groups. *P. vivax* = *Plasmodium vivax.*

A total of 447 febrile patient samples that were *P. vivax* smear positive were collected at 27 health facilities from seven major regions of Ethiopia: Afar, Amhara, Benishangul/Gumuz, Gambella, Oromia, Sidama, and Southern Nations Nationalities and People’s Region (SNNPR) (Figure 1) from 2020 to 2021. These seven regions vary in elevation. Afar is in the northeastern part of the country with an elevation of 379 m (latitude 11.568°N, longitude 41.438°E); Amhara is in the north with an elevation of 1,268 m (latitude 11.66334°N, longitude 38.821903°E); Benishangul/Gumuz is in the west with an elevation of 1,909 m (latitude 10.78°N, longitude 35.56578°E); Gambella is in the west, bordering Sudan, with an elevation of 447 m (latitude 8.24999°N, longitude 34.5833°E); Oromia is in the east with an elevation of 959 m (latitude 7.98906°N, longitude 39.38118°E); Sidama is in the southeast with an elevation of 1,742 m (latitude 6.7372°N, longitude 38.4008°E); and the SNNPR is in the south with an elevation of 1,200 m (latitude 6.05862°N, longitude 36.7273°E).

### Blood sample collection and microscopic detection of *Plasmodium* species.

Blood samples were collected by finger-pricking from 447 study participants (males, *n* = 269; females, *n* = 171; and missing information, *n* = 7) with at least two clinical symptoms, and who were suspected of having a malarial infection. Among the seven study regions of Ethiopia, the greatest proportion of the samples were from the SNNPR (34.8%), followed by Oromia (31.3%) and Amhara (23.9%). Afar (1.1%) and Sidama (0.4%) had the smallest sample size (Supplemental Table 1).

All samples were collected from *P. vivax* microscopy-confirmed patients. Thick and thin blood films were prepared for microscopic screening of *Plasmodium* parasites. Blood smears were stained for 10 minutes with 10% Giemsa staining solution (pH 7.2). The parasite species, developmental stages of the parasites, and density of asexual parasites and sexual gametocytes were examined using microscopy. A minimum of 200 microscopic fields were examined at ×1,000 magnification using oil immersion optics before a slide was declared negative for malaria parasites using the light microscope. The number of parasites per microliter of blood was estimated from the thick films as the number of parasites per 200 white blood cells multiplied by 8,000 (an average white blood cell count per microliter) and was then divided by 200. Slides were read twice by the primary readers at the site of the study, and the secondary readers at the Ethiopian Public Health Institute. Discordant results were confirmed by tertiary expert readers. Final species diagnosis was decided by the expert readers. Rapid diagnostic testing was also conducted for malaria detection.^19^^,^^20^ Dried blood spot (DBS) samples were collected for molecular screening of *Plasmodium* species.

### Molecular screening of *Plasmodium species.*

Parasite DNA was isolated from a DBS using the Saponin/Chelex method.^21^ *Plasmodium vivax* and *Plasmodium falciparum* were detected using the SYBR Green quantitative polymerase chain reaction (qPCR) detection method^2^ with the published primers (forward: 5′-GAATTTTCTCTTCGGAGTTTATTCTTAGATTGC-3′; reverse: 5′-GCCGCAAGCTCCACGCCTGGTGGTGC-3′) specific to *P. vivax*^22^^,^^23^ and *P. falciparum* 18S recombinant RNA (forward: 5′-AGTCATCTTTCGAGGTGACTTTTAGATTGCT-3′; reverse: 5′-GCCGCAAGCTCCACGCCTGGTGGTGC-3′).^24^ Amplification was conducted in a 20-*μ*L reaction mixture containing 2 *μ*L genomic DNA, 10 *μ*L SYBR Green qPCR Master Mix (Thermo Scientific), and 0.5 *μ*M primer. The reactions were performed using the QuantStudio Real-Time PCR Detection System (Thermo Fisher), with an initial denaturation at 95°C for 3 minutes, followed by 45 cycles at 94°C for 30 seconds, 55°C for 30 seconds, and 68°C for 1 minute, with a final 95°C for 10 seconds. This was followed by a melting–curve step of temperature ranging from 65 to 95°C with 0.5°C increments to determine the melting temperature of each amplified product. Each assay included positive controls of *P. vivax* Pakchong (MRA-342G) and Nicaragua (MRA-340G) isolates, *P. falciparum* isolates 7G8 (MRA-926) and HB3 (MRA-155), in addition to negative controls, including uninfected samples and water. A standard curve was produced from a 10-fold dilution series of the *P. vivax* and *P. falciparum* control plasmid to determine the amplification efficiency of the qPCR. Melting-curve analyses were performed for each amplified sample to confirm specific amplifications of the target sequence. The slope of the linear regression of threshold cycle (Ct) number versus log10 (gene copy number [GCN]) was used to calculate the amplification efficiency of each plate run based on internal standard controls. For the measurement of reproducibility of the Ct number, the mean Ct value and the standard error was calculated from three independent assays of each sample. A cutoff threshold of 0.02 fluorescence units that robustly represented the Ct at the log-linear phase of the amplification and above the background noise was set to determine the Ct value for each assay. Samples yielding Ct values >40 (as indicated in the negative controls) were considered negative for *Plasmodium* species. Parasite density in a sample was quantified by converting the Ct values into the GCN using the following equation: GCN_sample_ = 2*^E^*^×(40-Ct^ ^sample)^, where *E* stands for amplification efficiency. The differences in the log-transformed parasite GCN between samples among the study sites were assessed for significance at a level of 0.05.^25^

### Duffy blood group genotyping.

For all DBS samples, we used the qPCR-based TaqMan assay to examine the point mutation (c.1-67T>C; rs2814778) in the GATA-1 transcription factor binding site of the *DARC* gene. The following primers (forward: 5′-GGCCTGAGGCTTGTGCAGGCAG-3′; reverse: 5′-CATACTCACCCTGTGCAGACAG-3′) and dye-labeled probes (FAM-CCTTGGCTCTTA[*C*]CTTGGAAGCACAGG-BHQ; HEX-CCTTGGCTCTTA[*T*]CTTGGAAGCACAGG-BHQ) were used. Each PCR contained 5 *μ*L TaqMan Fast Advanced Master mix (Thermo Scientific), 1 *μ*L DNA template, 0.5 *μ*L of each primer (10 nM), and 0.5 *μ*L of each probe (10 nM). The reactions were performed with an initial denaturation at 95°C for 2 minutes, followed by 45 cycles at 95°C for 3 seconds and 58°C for 30 seconds. A no-template control was used in each assay. The *Fy* genotypes were determined by the allelic discrimination plot based on the fluorescent signal emitted from the allele-specific probes. For *P. vivax*–positive samples, a 1,100-bp fragment of the *DARC* gene was further amplified using previously published primers.^3^ Each PCR contained 20 *μ*L DreamTaq PCR Mastermix, 1 *μ*L DNA template, and 0.5 *μ*L of each primer. Conditions for the PCR were 94°C for 2 minutes, followed by 35 cycles of 94°C for 20 seconds, 58°C for 30 seconds, and 68°C for 60 seconds, followed by a 4-minute extension. The PCR products were purified and Sanger-sequenced. Chromatograms were inspected visually to determine and confirm the *Fy* genotypes based on the TaqMan assays.^25^

## STATISTICAL ANALYSES

SPSS v. 21.0 (SPSS Inc., Chicago, IL) was used for analyzing the sociodemographic information of the participants using descriptive statistics. To test the association between malaria infection and factors including sex, age, ethnicity, and clinical symptoms, bivariate and multivariate logistic regression were performed. The odds ratio (OR) and associated 95% CI were computed to assess the strength of association. *P*-values <0.05 were considered significant.

## RESULTS

### Distribution of the Duffy genotypes and prevalence of gametocytes across Ethiopia.

Of the 447 study participants, 421 (94.2%) were confirmed with having *Plasmodium* infections. Approximately 72% of the cases were *P. vivax* infections (322 of 447), 1.6% were *P. falciparum* infections (7 of 447), and 20.6% were *P. vivax–P. falciparum* mixed infections (92 of 447). Twenty of the 447 (4.5%) study participants were Duffy-negative. Of the 20 Duffy-negative participants, seven were infected with *P. vivax*, nine were infected with both *P. vivax* and *P. falciparum*, and four were not infected. Duffy-negative infections by *P. vivax* were observed in different sites across Ethiopia, specifically in the Amhara, Oromia, Benishangul/Gumuz, and SNNPR regions, but not in the Afar, Gambella, and Sidama regions. This could be a result of the small sample size in these study sites.

The gametocyte prevalence in Duffy-negative individuals was 31.3% (5 of 16), with one of them detected in a *P. vivax–P. falciparum* mixed infection. This proportion was not significantly different from the Duffy-positive samples (37.7%; 150 of 398), with 26 of them detected in *P. vivax–P. falciparum* mixed infections. Gametocyte stages of *P. vivax* infections were mostly found in the SNNPR (46.2%; 70 of 155) and Amhara (30.3%; 47 of 155), followed by Oromia (12.9%; 20 of 155). There were no gametocytes detected in *P. falciparum* infections. Five gametocyte-positive *P. vivax* infections were detected in Duffy-negative participants including two from Amhara, two from the SNNPR, and one from Oromia (Table 1).

**Table 1:** Distribution of the Duffy genotypes and gametocyte prevalence among *Plasmodium vivax*, *Plasmodium falciparum*, and mixed *P. vivax* and *P. falciparum* infections in the Ethiopian study participants

Region	Sample (*n*)	Duffy-Positive Sample					Duffy-Negative Sample				
		*Pv*	*Pf*	Mixed *Pv-Pf*	Malaria-Negative	*Pv* with Gametocytes	*Pv*	*Pf*	Mixed *Pv-Pf*	Malaria-Negative	*Pv* with Gametocytes
Afar	5	0	0	5	0	0	0	0	0	0	0
Amhara	107	65	4	18	15	45	5	0	0	0	2
Benishangul/Gumuz	22	6	0	13	0	5	0	0	2	1	0
Gambella	15	7	0	8	0	11	0	0	0	0	0
Oromia	140	115	3	13	1	19	1	0	4	3	1
Sidama	2	2	0	0	0	2	0	0	0	0	0
SNNPR	156	120	0	26	6	68	1	0	3	0	2
Total	447	315	7	83	22	150	7	0	9	4	5

*Pf* = *Plasmodium falciparum*; *Pv* = *Plasmodium vivax*; *Pv-Pf* = mixed *P. vivax* and *P. falciparum* infections; SNNPR = Southern Nations, Nationalities, and People’s Region.

### Asexual parasitemia and parasite stage comparisons.

**Figure 2.**
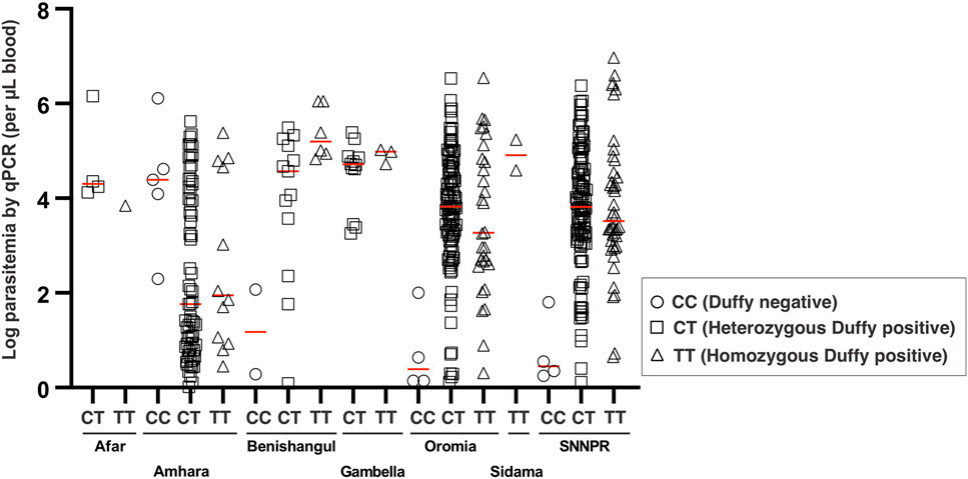
Parasitemia comparison by quantitative polymerase chain reaction (qPCR) among Duffy-positive (CT = heterozygous Duffy positive; TT = homozygous Duffy positive) and Duffy-negative (CC) patients with malaria in different major regions of Ethiopia. No significant difference (*P* >0.05) was observed between heterozygous and homozygous Duffy positive infections. The sample size of Duffy-negative infections was limited for statistical testing. SNNPR = Southern Nations, Nationalities, and People’s Region.

No significant difference was detected in parasitemia among the *P. vivax* samples collected from southwestern, southern, and eastern regions of Ethiopia, except for samples in Amhara (northwest of Ethiopia), which showed lower parasitemia among homozygous and heterozygous Duffy-positive samples. Although previous studies indicated that parasitemia in Duffy-negative individuals are expected to be low, our data show that *P. vivax* parasitemia in Duffy-negative samples widely varied among infections, with relatively low parasitemia observed in Oromia and the SNNPR, but higher in Amhara (Figure 2).

**Figure 3.**
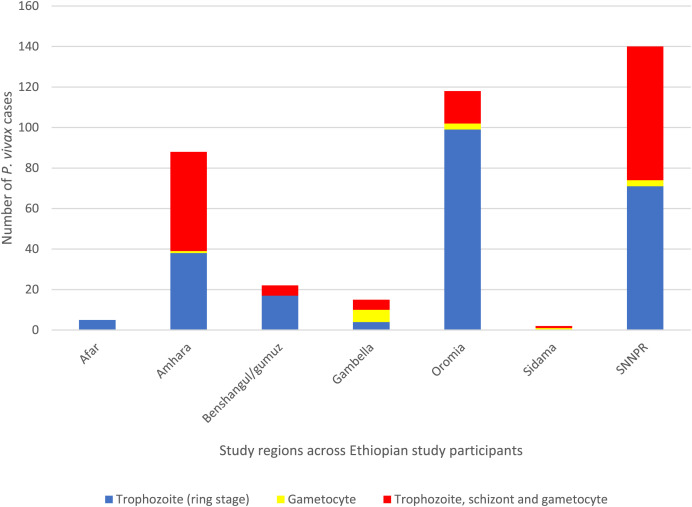
Comparison of parasite stages of *Plasmodium vivax* (*P. vivax*)–infected samples by microscopic examination across Ethiopian study participants from broad regions.

Most of the infections had mixed parasite stages, and the proportion of parasite stages varied among regions. In the SNNPR, 71 of 140 (50.7%) *P. vivax* samples had trophozoites; 66 (47.1%) had mixed trophozoite, schizont, and gametocyte stages; and three (2.1%) had gametocytes only. In Oromia, 99 of the 118 (83.9%) *P. vivax* samples had trophozoites; followed by 16 (13.6%) with mixed trophozoite, schizont, and gametocyte stages; and three (2.5%) with gametocytes only. In Amhara, a similar proportion was observed, where 44 of 85 (51.8%) *P. vivax* samples had mixed trophozoite, schizont, and gametocyte stages; followed by 40 (47.1%) with trophozoites; and one (1.2%) with gametocytes. In Benishangul/Gumuz, 16 of 21 (76.2%) *P. vivax* samples had trophozoites and five (23.8%) had mixed trophozoite, schizont, and gametocyte stages. In Gambella, 4 of 15 (2.7%) *P. vivax* samples had trophozoites and 11 of 15 (73.3%) had trophozoites mixed with gametocytes. In Afar, all five mixed *P. vivax* and *P. falciparum* samples had trophozoites. In Sidama, the two *P. vivax* samples had mixed trophozoite, schizont, and gametocyte stages. Overall, almost all samples had trophozoites and mixed stages across study sites (Figure 3).

**Figure 4.**
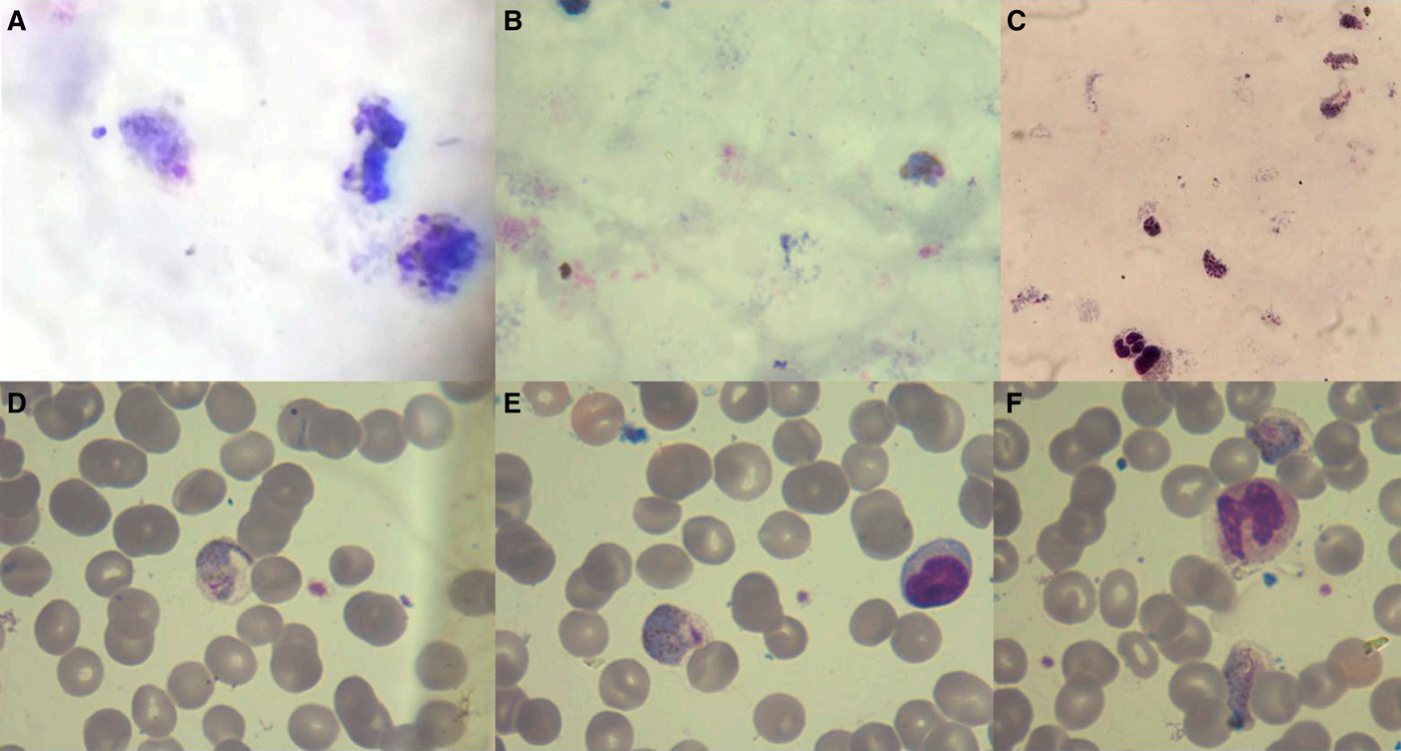
Microscopic images of Giemsa-stained thick (**A–C**) and thin (**D–F**) smears showing different development stages of *Plasmodium vivax* in Duffy-negative infections. (**A, B**) Gametocytes, (**C**) gametocytes and early trophozoites, and (**D–F**) early- and late-stage gametocytes.

**Figure 5.**
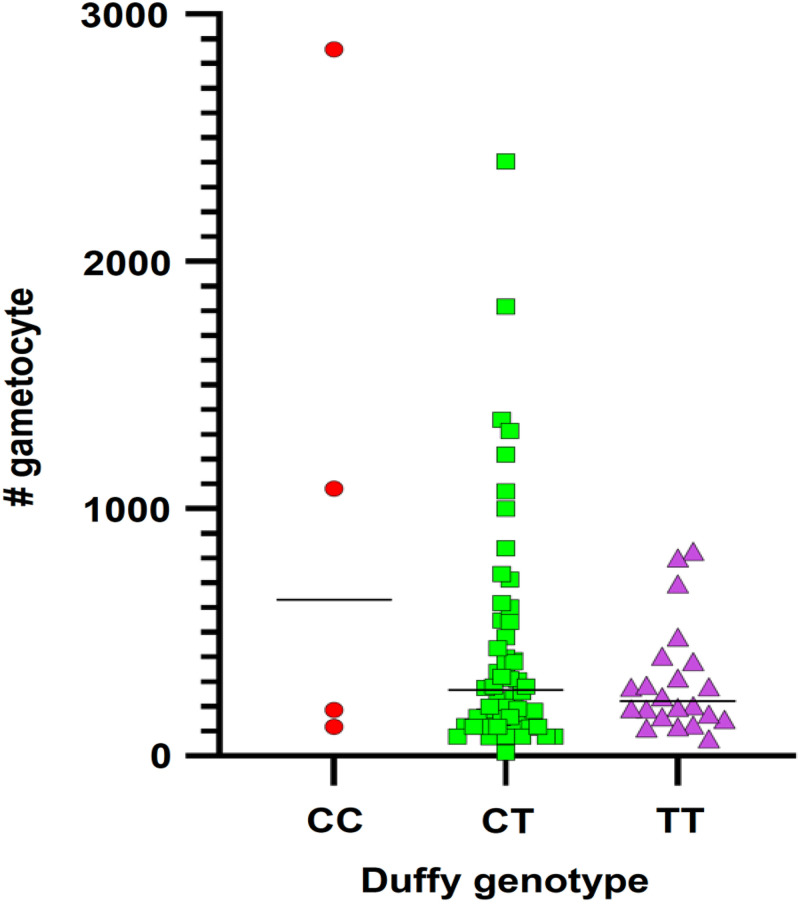
The gametocyte counts among homozygous Duffy-negative (CC), heterozygous Duffy-positive (TC) and homozygous Duffy-positive (TT) isolates of *Plasmodium vivax* species.

In the Duffy-negative infections, gametocytes with mixed trophozoite stages of *P. vivax* were observed using microscopy (Figure 4). The gametocyte counts of *P. vivax* was done with a light microscope against 200 white blood cells (WBCs) and was calculated using an average WBC count per microliter of blood. The highest gametocyte count was 2,856 gametocyte/*μ*L, detected in homozygous Duffy-negative individuals and the lowest gametocyte count was 15 gametocytes/*μ*L detected in heterozygous Duffy-positive individuals. The average number of *P. vivax* gametocytes among all gametocyte-positive samples was 449 gametocytes/*μ*L blood, of which the average gametocyte counts of homozygous Duffy-negative samples was 1,060 gametocytes/*μ*L, of heterozygous Duffy-positive samples was 425 gametocytes/*μ*L, and of homozygous Duffy positive samples was 395 gametocytes/*μ*L (Figure 5).

### Duffy blood group and other factors associated with *Plasmodium* infections.

The bivariate analysis was done to show the association of *P. vivax* infection with independent factors. The prevalence of *P. vivax* infections in Duffy-positive individuals was about four times more likely than in patients who were Duffy negative (OR = 4.6, 95% CI = 1.4–14.96, *P =* 0.011). *Plasmodium vivax* infection was not significantly different among males and females. Although the prevalence of *P. vivax* infection was recorded in all age groups, a relatively greater prevalence was seen in the age group younger than 15 years, and was three times more likely than the age group older than 45 years (OR = 2.9, 95% CI = 0.45–1.98, *P =* 0.98). The *Plasmodium* infections were significantly different among various clinical symptoms of the study participants. The odds of infection among patients with headache were three times more likely than without headache (OR = 3.0, 95% CI = 0.81–11.14, *P =* 0.09), among patients who indicated sweating as a symptom were three times more likely to be infected than those who did not (OR = 3.5, 95% CI = 1.7–7.44, *P =* 0.0009), and among patients with chills were more than two times more likely than those without chills (OR = 2.5, 95% CI = 1.05–4.8, *P =* 0.037). No significant difference was found in malaria symptoms such as fever, muscle and joint pain, and nausea and vomiting between *Plasmodium*-infected and uninfected individuals (*P* >0.05) (Table 2).

**Table 2:** Results of bivariate odds ratio to determine the main predictors of *Plasmodium* infections across the Ethiopian study participants*

Parameter	Infection Rate by 18S qPCR			
	Total Sample (*N*)	Infected (*n*)	Not Infected (*n*)	Odds Ratio (95% CI), *P*-Value
Duffy status				
Duffy positive	427	405	22	4.6 (1.4–14.96), *P =* 0.011^†^
Duffy negative	20	16	4	1
Sex				
Female	171	159	12	1
Male	269	249	20	0.94 (0.45–1.98), *P =* 0.87
Age (years)				
≤15	150	143	7	2.9 (0.55–15.4), *P =* 0.21
>15 and <45	273	250	23	1.55 (0.33–7.26), *P =* 0.58
≥45	16	14	2	1
Symptom				
Fever				
Yes	398	369	29	0.59 (0.03–10.43), *P =* 0.72
No	10	10	0	1
Headache				
Yes	391	365	26	3.0 (0.81–11.14), *P =* 0.099
No	17	14	3	1
Fatigue				
Yes	257	238	19	0.89 (0.4–1.96), *P =* 0.77
No	151	141	10	1
Muscle and joint paint				
Yes	257	240	17	1.12 (0.52–2.41), *P =* 0.77
No	163	151	12	1
Chills				
Yes	258	245	13	2.25 (1.05–4.8), *P =* 0.037^†^
No	150	134	16	1
Sweating				
Yes	218	208	10	3.5 (1.7–7.44), *P =* 0.001^†^
No	200	171	29	1
Nausea and vomiting				
Yes	177	163	14	0.81 (0.38–1.73), *P =* 0.59
No	230	215	15	1

qPCR = quantitative polymerase chain reaction.

* The discrepancy in the number of samples in the analyses was the result of missing data in clinical symptoms.

^†^ Significant at 0.05.

## DISCUSSION

In sub-Saharan Africa, where Duffy-negative individuals are predominant, *P. vivax* malaria has been reported, but whether these infections can transmit among individuals is poorly documented. Our study indicates that *P. vivax* infections in Duffy-negative individuals are distributed across broad regions of Ethiopia. The prevalence of *P. vivax* among Duffy-negative participants was 3.8% (16/421). This result is in line with previous studies in the country that revealed a prevalence of *P. vivax* among Duffy-negative individuals of 2.9%^26^ and 4.4%.^27^ On the contrary, our result is less than that found in Sudan (17.9%).^28^ In the general populations, Duffy negativity varies from 20% to 36% in East Africa to 84% in Southern Africa.^4^ The average parasite density in Oromia and the SNNPR (southern Ethiopia) was low among Duffy-negative individuals. This was consistent with our previous findings^4^ that showed an overall lower parasite density in Duffy-negative than Duffy-positive infections collected from Jimma and Bonga. Nonetheless, in Amhara, we found a few Duffy-negative *P. vivax* infections with relatively high parasitemia, suggesting certain *P. vivax* strains can invade and replicate efficiently in Duffy-negative erythrocytes. In addition, variations in host immune responses and environments across different regions may contribute to differences in parasitemia among Duffy-negative infections. The exact mechanisms of Duffy-negative erythrocyte invasion by *P. vivax* are still unclear and merit further investigation. For instance, *P. vivax* glycosylphosphatidylinositol-anchored micronemal antigen and *P. vivax* merozoite surface protein 1 paralog have recently been shown^29^^–^^31^ to bind to both Duffy-positive and Duffy-negative red blood cells (RBCs), suggesting their possible involvement in a Duffy-independent invasion pathway. The *P. vivax* reticulocyte binding protein 2b of *P. vivax* has been shown^5^^,^^32^ to bind to transferrin receptor 1 to invade Duffy-positive RBCs, and thus present alternative pathways for Duffy-negative erythrocyte invasion. Such findings are critical to the development of blood-stage vaccines against the parasites.^33^^,^^34^

Among regions, the difference in *P. vivax* gametocyte production in Duffy-positive and Duffy-negative individuals was not significant. However, the mean number of gametocytes count among Duffy-negative participants was higher compared with Duffy-positive participants, despite a small number of Duffy-negative samples with gametocytes. This result indicates the dominance of sexual stages of *P. vivax* parasites which can be a signal for the existence of asymptomatic infections in Duffy-negative individuals. The detection of *P. vivax* gametocytes in Duffy-negative infections in Amhara, Oromia, and the SNNPR raises concerns that these infections not only cause clinical symptoms, but also contribute to transmission. A recent study^16^ on gametocyte infectivity by membrane feeding experiments in Adama, Ethiopia, showed that homozygous Duffy positive individuals with high parasitemia were more subject to infection from *Anopheles* mosquitoes, but heterozygous Duffy-positive individuals with high gametocytemia had a low infection rate. This result warrants further study to determine and compare the transmissibility of *P. vivax* among Duffy genotypes based on membrane feeding experiments beyond the sexual and asexual parasite count.

In Ethiopia, Duffy-negative and Duffy-positive individuals coexist, but the extent of transmission remains uncertain. It is possible that the asexual parasites converted into gametocytes and spread from Duffy-negative to other Duffy-negative or Duffy-positive individuals.^26^^,^^35^ This finding lends support to an earlier study^4^ that showed the parasites detected in Duffy-negative and Duffy-positive populations were not genetically different. Based on computation modeling, Duffy-negative populations in Ethiopia can serve as both the source and sink of infections, although transmission is likely more frequent in Duffy-positive populations.^4^^,^^5^ Given that *P. vivax* has been widely reported in West and Central Africa, where >90% of the populations are Duffy-negative, these infections can certainly serve as reservoirs for transmission both at the local and regional levels.^5^^,^^27^ As a result of being exposed previously, the host may have acquired immunity against symptomatic blood-stage parasitemia; however, because of the early gametocyte development of *P. vivax*, long-lasting subclinical illnesses may still contribute to continuous transmission.^36^^,^^37^ In our study, all gametocytes detected among the mixed infections were *P. vivax*. This result supports the notion that the development of *P. vivax* gametocytes is much faster than that of *P. falciparum* at the onset of symptoms in febrile patients with malaria, and that *P. falciparum* gametocytes are detected seldomly in routine microscopic examination of febrile patients with malaria.^38^

Most *P. vivax* infections in Amhara, Gambella, Sidama, and the SNNPR had mixed parasite stages including gametocytes, whereas in Oromia and the SNNPR, trophozoites were prominent in most samples. This variation in parasite developmental stages could be associated with environmental, host, and parasite factors among different study districts. The epidemiology of malaria within each district may also be a determining factor. For instance, although the general proportion of *P. falciparum* and *P. vivax* in Ethiopia is 60% and 40%, respectively, considerable regional differences exist.^39^ Warmer temperatures and greater rainfall/humidity in lowland rather than highland areas may allow parasites to develop faster and produce greater numbers of gametocytes, which result in majority mixed stages among *P. vivax* infections and enhance transmission. This might be supported by the ability of the parasite to develop the asexual stage into gametocytes faster, within 48 hours after generation of the first merozoites in the blood. The flexible nature of *P. vivax*–infected and swollen RBCs resulted from gametocyte development helps to stay all stages of the infections together in the peripheral blood.^13^ The unique biological features and genetic variability of the *P. vivax* parasites certainly present a challenge in eradicating malaria in Ethiopia.^40^^,^^41^

For all *P. vivax*–confirmed infections, typical symptoms were fever as well as headache and fatigue. Other symptoms including muscle and joint pain, chills, sweating, and vomiting vary by individual across the seven study regions. Interestingly, our analyses revealed that *P. vivax* cases were more likely to occur in individuals 15 years or younger followed by those older than 15 and younger than 45 years. Such a demographic pattern could be explained if the host immunity in old individuals is higher compared with younger adults and children. Mosquito vector feeding time and behavior (outdoor or indoor resting/biting), individuals’ occupation (outdoor or indoor), the environment (rural or remote populations), and economic status (poverty) may also contribute.^42^ These factors are critical when identifying disease trends or at-risk populations.^5^

## CONCLUSION

The prevalence of Duffy-negative individuals among patients with *P. vivax* malaria varies across Ethiopia. Our study confirms that Duffy negativity does not protect completely against *P. vivax* infection, and these infections are frequently associated with low parasitemia, which may represent hidden reservoirs that can contribute to transmission. Understanding *P. vivax* transmission biology and gametocyte function via infectivity studies and in vitro assays, especially in Duffy-negative populations, will enhance the treatment and control strategies of *P. vivax* malaria in Africa. Further study is needed to quantify *Plasmodium vivax surface protein* (*Pvs*25) transcripts by quantitative reverse transcription-PCR for gametocyte density in Duffy-negative infected samples and to expand sample size to allow for fair comparisons of gametocyte carriage between Duffy-positive and Duffy-negative infections. A deeper comprehension of the association between Duffy negativity and the invasion processes of *P. vivax* would aid the development of *P. vivax*–specific eradication tactics.

## Supplemental Materials

10.4269/ajtmh.23-0877 Supplemental Materials

## Data Availability

All data produced in our study are available upon reasonable request made to the corresponding author per institutional and national legal norms and procedures.
