# Supplementary material for: Prevalence and Characteristics of Plasmodium vivax Gametocytes in Duffy-Positive and Duffy-Negative Populations across Ethiopia
Source: Am J Trop Med Hyg. 2024 Apr 16;110(6):1091–9. doi: 10.4269/ajtmh.23-0877 (PMC11154031; doi:10.4269/ajtmh.23-0877)
Supplement: Supplemental Materials [file tpmd230877.SD1.pdf]

Supplementary table 1. Socio-demographic characteristics of the study participants in seven regional states across Ethiopian study participants.

**Characteristics**		**Number of participants (%)**
**Gender**		
	Male	269 (60.1%)
	Female	171 (38.2%)
**Age (years old)**		
	< 15	150 (33.5%)
	≥ 16 and < 45	273 (61.0%)
	≥ 45	16 (3.5%)
**Region**		
	Afar	5 (1.1%)
	Amhara	107 (23.9%)
	Benishangual/Gumuz	22 (4.9%)
	Gambella	15 (3.3%)
	Oromia	140 (31.3%)
	Sidama	2 (0.4%)
	SNNPR	156 (34.8%)
